# Automated indexing using NLM's Medical Text Indexer (MTI) compared to human indexing in Medline: a pilot study

**DOI:** 10.5195/jmla.2023.1588

**Published:** 2023-07-10

**Authors:** Eileen Chen, Julia Bullard, Dean Giustini

**Affiliations:** 1 eileen.0415@livemail.tw, Student, University of British Columbia, School of Information, Vancouver, British Columbia, Canada.; 2 julia.bullard@ubc.ca, Assistant Professor, University of British Columbia, School of Information, Vancouver, British Columbia, Canada.; 3 dean.giustini@ubc.ca, Librarian, University of British Columbia, Biomedical Branch Library, Vancouver General Hospital, Vancouver, British Columbia, Canada.

**Keywords:** Automated indexing, human indexers, information retrieval, Medical Text Indexer (MTI), Medline, PubMed.

## Abstract

**Objective:**

In 2002, the National Library of Medicine (NLM) introduced semi-automated indexing of Medline using the Medical Text Indexer (MTI). In 2021, NLM announced that it would fully automate its indexing in Medline with an improved MTI by mid-2022. This pilot study examines indexing using a sample of records in Medline from 2000, and how an early, public version of MTI's outputs compares to records created by human indexers.

**Methods:**

This pilot study examines twenty Medline records from 2000, a year before the MTI was introduced as a MeSH term recommender. We identified twenty higher- and lower-impact biomedical journals based on Journal Impact Factor (JIF) and examined the indexing of papers by feeding their PubMed records into the Interactive MTI tool.

**Results:**

In the sample, we found key differences between automated and human-indexed Medline records: MTI assigned more terms and used them more accurately for citations in the higher JIF group, and MTI tended to rank the Male check tag more highly than the Female check tag and to omit Aged check tags. Sometimes MTI chose more specific terms than human indexers but was inconsistent in applying specificity principles.

**Conclusion:**

NLM's transition to fully automated indexing of the biomedical literature could introduce or perpetuate inconsistencies and biases in Medline. Librarians and searchers should assess changes to index terms, and their impact on PubMed's mapping features for a range of topics. Future research should evaluate automated indexing as it pertains to finding clinical information effectively, and in performing systematic searches.

## INTRODUCTION

The Medical Text Indexer (MTI) is an automated indexing tool developed by the U.S. National Library of Medicine (NLM) and the Lister Hill National Center for Biomedical Communications. In 2002, the MTI was introduced to partly automate the indexing of Medline citations. NLM has developed newer versions of MTI since then, and moved to fully automated indexing by mid-2022 [1]. The latest version is the MTI-Auto (MTIA), which draws on PubMed's Medical Subject Headings (MeSH) mapping and Related Citations feature [1]. Today, MTI's goal is to supply ranked lists of MeSH descriptors, supplementary concepts, and publication types for new records [1]. Human curation of Medline records is now focused on quality assessment of select citations of “genes and proteins, cases of known ambiguity, clinical trials” and “[r]andom sets of citations” [1].

Since 2002, researchers have tested MTI's precision and recall [2–3], but few publicly available studies have examined its performance compared to human indexing. A 2023 study comparing MTI's performance to human indexing found that MTI's reliability at identifying diseases in grant descriptions, patent claims, and drug indications was comparable to that of human indexers [4]. However, this level of accuracy does not apply to all topics, as suggested by another recent study that found that MTI was weak at predicting terms that were less common in the corpus of available biomedical literature [5]. Both papers fall outside of health librarianship literature but reveal insights on how automated indexing is viewed through scientific disciplines.

According to the National Information Standards Organization (NISO)'s Criteria for Indexes, indexing terms should be “usable and intuitive,” and promote “content findability and discoverability” [6]. While the usability and intuitiveness of MTI indexing from a searcher's perspective is largely unexplored, NLM aims to make indexing more efficient by mapping large quantities of text to relevant MeSH quickly and by using machine learning to retrieve probabilistically and semantically likely terms [1].

Machine learning algorithms tend to perpetuate biases of various kinds [7]. Studies pre-dating automated MTI indexing found that in fields such as alternative medicine and chronobiology, for example, Medline indexing was often inconsistent, inadequate or both [8–10]. MTI's reliance on statistical methods and ongoing expansion into machine learning may cause it to inherit problems and biases in existing datasets.

MTI tends to perform better on papers that conform to specific conventions. For example, MTI is programmed to recommend MeSH terms based on the titles and abstracts of papers and performs better when study populations are clearly defined [2] or when abstracts are divided into sections that follow a standard Introduction, Methods, Results, and Discussion (IMRaD) format [11]. Abstracts extracted from a publication type that differs or deviates from this format may receive suboptimal indexing.

In this study, we explore the strengths and weaknesses of automated indexing by using the only publicly available MTI from 2011 and examine patterns reflecting age and gender biases. As a pilot study, we lay the foundation for future comparisons of indexing quality for bibliographic databases that plan the shift toward automated methods. While our study does not cover each biomedical topic fully, we provide a basis for future research of the impact of automated indexing. Our aim is to help health information professionals understand the impact of automated indexing of biomedical literature, and how it may affect information retrieval in tools such as PubMed (Medline).

## METHODS

We conducted our pilot study by creating a sample of 20 Medline citations published in the year 2000. All research materials, including a glossary of terms, our datasets, and preliminary presentations are available on Open Science Framework [12]. Citations were selected from journals listed in the Abridged Index Medicus (AIM), a subset of core clinical journals in Medline that the NLM discontinued in 2020. We chose to use AIM's list for our sample due to its broad coverage and the availability of complete MeSH records for papers published. We chose the year 2000 because it was a full year before MTI was introduced, ensuring that the human indexing lists we used were not influenced by MTI.

We ranked each AIM journal using Clarivate's Journal Citation Reports (JCR) and its trademarked Journal Impact Factor (JIF) and selected ten journals with the highest and ten with the lowest 2020 JIFs for a total of 20 journals. The JIF is defined as the sum of citations received in a given year for a journal's previous two years of publications divided by the number of citable items published in that journal in the previous two years and is commonly interpreted as an indicator of a journal's influence or quality in its field [13–14]. We used this measure to identify higher- and lower-JIF journals, and to compare the depth and quality of indexing in each group. We used filters to select five citations from each quarter of the year. Results were sorted by PubMed's relevance ranking by default, and the first citation in the list was selected. Any papers that lacked a Medline record or abstract were removed.

The free Interactive MTI allows users to generate MeSH terms for a body of text under 10,000 characters and includes several filtering, post-processing, output, and debug customizations [15]. We copied the titles and abstracts into the text box of the Interactive MTI, and made the following changes to settings:

- Filtering: default for non-Medline citations; MTI as First Line Indexer (MTIFL)
- Post-processing: default
- Output: Just the Facts (first run) / Full Listing w/ Detailed (second run)
- Debug: none

In 2011, the first line indexer tool (MTIFL) was developed and was later replaced by the MTIA. We were unable to obtain access to the MTIA and used the MTIFL setting as the only publicly available option. We adjusted each setting alongside the MTIFL to simulate our understanding of the automated indexing process. As new citations awaiting indexing will not have PMIDs as a basis for retrieving terms from PubMed Related Citations, filtering was set to “default for non-MEDLINE citations,” which does not find PMIDs.

The ranked terms produced by Just the Facts (JTF) are a list of index terms recommended in priority order as output for a given text. For each output, the list displays the PMID if applicable, indexing term, Concept Unique Identifier (CUI) (a code identifying a concept's unique meaning in the Unified Medical Language System), and score. Scoring is a complex process based on the detection and analysis of co-occurring terms, related citations, and path weighting [16]. The list includes main headings, check tags, subheadings, and supplemental concepts. Here, we selected the JTF as the basis for comparing MTI and human indexing lists, as it is a shorter list that more closely represents MTI's final choices.

The longer ranked list, called the Full Listing, includes index terms that MTI retrieves based on text inputs. Each entry includes a PMID (if applicable), list position, term, CUI, score, type, misc., location, and paths. “List Position” is the term's rank in the Full Listing, “Type” refers to the classification of a term (e.g. main heading, check tag), “Misc” identifies term replacements if an entry term differs from a preferred term, “Location” clarifies whether a term was found in the title, abstract, or both, and “Paths” describes the pathway(s) MTI took to retrieve the term (i.e. MetaMap, PubMed Related Citations, or John Wilbur's Trigram Method) [17]. We referred to the Full Listing to find terms used by human indexers that were not in the JTF list.

The following information was collected for each citation:

- PMID: PMID of the citation
- Subject area(s): subject area(s) of source journal, according to JCR
- Journal: title of the source journal containing the citation
- Month: month of the citation's publication
- Title and abstract word count: total word count of text entered into Interactive MTI, consisting of the citation's title and abstract
- Total MTI terms: total number of terms in the JTF list
- Total human terms: total number of terms used by the human indexer, not counting terms with multiple subheadings as separate entries
- Total identical terms: total number of terms that were identical between the human indexer and MTI
- MTI only: number of terms in the JTF list that were not used by the human indexer
- Human only: number of terms in the human indexer's list that were not used by MTI
- Total Full Listing: number of terms in the Full Listing, including check tags (usually separately ranked, but part of the same list)
- Shared terms list: a list of identical terms used by MTI and the human indexer in ranked order from the Full Listing. Major headings were marked with an asterisk, and terms considered to be check tags by MTI were marked with an obelisk
- MTI only terms list: list of terms in JTF not used by the human indexer in ranked order. MeSH that did not exist in 2000 were marked with two asterisks
- Human only terms in Full Listing: list of terms in the human indexer's list not used by MTI in the JTF list, in order of their ranking in MTI's Full Listing. Major headings were marked with an asterisk, and terms considered check tags by MTI were italicized. Terms missing from the Full Listing were put at the end, in bold.

We examined the data collected to compare similarities and differences between automated processes of MTI and human indexers by considering:

- Total number of index terms between MTI and human indexers;
- How indexing compared between higher and lower JIF journals;
- Term selection between MTI and human indexers;
- Patterns and anomalies in check tags;
- Use of Male † and Female † check tags;
- Coverage of human indexing before and after considering synonymous terms;
- Coverage of human indexer only terms and instances where they were absent in Full Listing;
- Instances where MTI covered human indexer only terms in JTF list using synonyms
- Relationships between synonyms used by MTI and original terms.

## RESULTS

### Number of Index Terms

Our dataset reveals differences in MTI's confidence between higher and lower JIF journals, which is reflected in the total terms selected for the JTF list. However, we observed no relationship between the number of terms in the JTF list and words in a citation's title and abstract.

Tables 1 and 2 compare the mean and median numbers of MTI terms and human terms for citations from higher-and lower-JIF journals.

**Table 1.** Total numbers of MTI terms and human-indexed terms for each citation in a higher JIF journal.

Citation Number	Journal in which citation was published	Total MTI Terms	Total Human Terms
1	CA: a cancer journal for clinicians	12	22
2	New England journal of medicine	11	11
3	Lancet (London, England)	26	4
4	JAMA	21	13
5	Circulation	19	16
6	Annals of internal medicine	21	10
7	Journal of the American College of Cardiology	11	4
8	Blood	21	14
9	Gut	9	11
10	Gastroenterology	15	30
Mean (sd)		16.6 (5.7)	13.5 (7.9)
Median (IQR)		17 (11-21)	12 (10-16)

**Table 2.** Total numbers of MTI terms and human-indexed terms for each citation in a lower JIF journal.

Citation Number	Journal in which citation was published	Total MTI Terms	Total Human Terms
11	Journal of family practice	14	16
12	Southern medical journal	8	7
13	Clinical pediatrics	16	13
14	Nursing clinics of North America	4	13
15	Journal of laryngology and otology	7	8
16	Annals of otology, rhinology, and laryngology	17	13
17	Journal of nursing administration	5	6
18	Medicine	12	12
19	Journal of oral and maxillofacial surgery	8	9
20	American journal of physical medicine & rehabilitation	11	15
Mean (sd)		10.2 (4.5)	11.2 (3.5)
Median (IQR)		9.5 (7–14)	12.5 (8–13)

The average number of terms in the JTF list for citations in higher JIF journals was 16.6 and the average for citations in lower JIF journals was 10.2, a difference of 6.4 terms. The total of human-indexed terms was higher for citations in higher JIF journals (13.5) compared to citations in lower JIF journals (11.2); however, the mean difference was 2.3. The median number of human-indexed terms was more consistent: 12 for citations in the higher JIF journals and 12.5 for citations in the lower JIF journals. The median number of MTI-indexed terms was 17 for citations in higher JIF journals, and 9.5 for citations in lower JIF journals, a difference of 7.5 terms.

### Comparison of Citations with High and Low Numbers of MTI Terms

The highest total of MTI terms for any citation was 26, followed by 21, as follows: Lancet (26 terms), JAMA (21 terms), Annals of Internal Medicine (21 terms), and Blood (21 terms). The first three journals were categorized in the broad subject “Medicine, general and internal” on JCR, and Blood was in “Hematology.”

The lowest number of terms were seen in Citations #4, #5, and #7 from the Nursing Clinics of North America (4 terms), Journal of Nursing Administration (5 terms), and Journal of Laryngology and Otology (7 terms) [18–20]. The first two fall under the subject area “Nursing,” while the third falls under “Otorhinolaryngology”. Table 3 summarizes the numbers of MTI and human indexer terms for these citations.

**Table 3.** Ranked list of highest and lowest number of MTI terms for seven citations, number of human indexer terms, and percentage of human terms covered in the JTF list.

Citation #	Journal	Number of MTI Terms	Number of Human Indexer Terms	Human Indexer Terms Covered by MTI (%)*
3	*Lancet*	26	4	100.00%
4	*JAMA*	21	13	30.77%
6	*Annals of Internal Medicine*	21	10	80.00%
8	*Blood*	21	14	57.14%
14	*Nursing Clinics of North America*	4	13	30.77%
15	*Journal of Laryngology and Otology*	7	8	50.00%
17	*Journal of Nursing Administration*	5	6	33.33%

* This adjusted percentage includes synonyms of human-indexed terms used by MTI, defined as terms located within 2 levels on the MeSH tree, or alternative terms listed in any of the Entry Term/See Also/Previous Indexing fields.

Citations with the highest number of MTI terms were from the higher JIF list, while citations with the lowest number of MTI terms were from the lower JIF list. The following sections analyze the suitability of MTI terms and discuss human indexer and MTI differences in Citations #3, #14, and #17.

### Citation #3: Hypertensive Emergencies

Vaughan and Delanty's paper discusses hypertensive emergencies by outlining risk factors, pathophysiological mechanisms, and therapies [21]. All 26 terms were mapped to sources in the title and abstract, including pathological manifestations (e.g. “Seizures”; “Edema”), risk factors (e.g. “Eclampsia”; “Pre-Eclampsia”), pathophysiology (e.g. “Up-regulation”; “Vasoconstrictor Agents”) and therapies (e.g. “Magnesium”; “Nitroprusside”). Nuances were seen, such as the choice to include “Occipital Lobe” but not “Parietal Lobe” and using “Dopamine” rather than the more context-specific “Dopamine Agonists”.

Three terms that MTI shared with the original human indexer were outside the top ten terms in the JTF list (“Antihypertensive Agents” (ranked 14); “Hypertension” (16); “Hypertensive Encephalopathy” (17)). The top terms included several types of therapies, and risk factors “Eclampsia” and “Pre-Eclampsia”, but none can be considered the focus or major topic of the papers.

### Citation #14: Genetic Counseling and Testing: Implications for Clinical Practice

This Nursing Clinics of North America paper by Johnson and Brensinger included a short abstract describing complex social and emotional concerns in genetic counseling and testing [22]. The citation generated four MTI terms while the original indexer applied 13 MeSH terms in total. All four MTI terms were also used by the human indexer (“Humans†” (0); “Genetic Counseling” (1); “Genetic Testing” (2); “Informed Consent” (3)). Of the human-indexed terms not included, five were check tags that were not mentioned in the abstract (“Child†” (22); “Adult†” (24); “Pregnancy†” (27); Infant, Newborn† (50); Female† (58)), and three invoked concepts not discussed in the abstract (“Prenatal Diagnosis” (7); “Medical History Taking” (10); “Prejudice” (15)).

The MeSH term “Ethics, Nursing” (20) was omitted. This nursing paper addresses “ethical and social concerns” associated with the use of genetic services. The 83-word abstract emphasizes the complexity of medical, emotional, ethical, and social issues of genetic counseling and testing. Despite the human indexer identifying these as key subjects, MTI only touched on ethics with “Informed Consent” (3) and did not include additional terms to address the implications of genetic services. “Prejudice” (15) and “Ethics, Nursing” (20) both had low rankings in the Full Listing.

### Citation #17: The Relationship of Nursing Practice Models and Job Satisfaction Outcomes

This Journal of Nursing Administration paper by Upenieks produced the same number of MTI (5 terms) as human-indexed terms (5 terms) [23]. The paper is about the effects of nursing practice models on outcomes, summarizes their benefits, and their reliance on good management. The terms MTI shared with the original human indexer were the check tag “Humans†” (0) and the heading “Job Satisfaction” (1). Three major headings and one check tag were left out, namely “Models, Nursing” (5); “Nursing” (8); “Outcome Assessment, Health Care” (31); and “United States†” (53). Of these, only the geographical location “United States” was absent from the abstract. The three major headings were covered in the title and abstract.

MTI added “Social Responsibility”; “Climate Change”; and “Attention”. The first two MeSH terms were listed in the abstract as subjects that nurses were aware of due to practice models. Both were ranked more highly than the missing major headings. The MeSH term “Climate Change” was unavailable to the original indexer in 2002 as it was introduced in 2010.

### Comparison of Male/Female Check Tag Rankings

Eighteen of the 20 citations deal with human populations. MTI identified “Humans†” as a check tag, ranking it at the top. Six citations were originally indexed by a human indexer with both “Male†” and “Female†”. MTI missed or misapplied age and sex check tags in the JTF list, and consistently ranked “Male†” before “Female†”.

In the Full Listing, MTI ranked check tags and main headings separately, with tags placed at the top. Some check tags are misidentified as main headings. Correctly identified check tags that are separately ranked show up in the JTF list. The difference of 3 in Citations #1, 2, and 16 reflect MTI's correct use of “Male†” and “Female†”, ranking them in a specific list, while differences in rank of 35, 48, and 61 positions reflect that MTI ranked the check tags among major headings in the Full Listing for Citations #13, 18, and 20 [24–29].

Table 4 compares rankings of each check tag in the citations, highlighting a gap between rankings of “Male†” and “Female†” in the Full Listing, with a mean difference of 25.5 places.

**Table 4.** Comparison of rankings of Male and Female check tags in six citations where both are used

Citation Number	Male	Female	Difference (F - M)
1	**0^†^**	**3^†^**	3
2	5^†^	8^†^	3
13	15	50	35
16	**1^†^**	**4^†^**	3
18	12^†^	60^†^	48
20	**9^†^**	**70^†^**	61
Mean	7	32.5	25.5

Bolded = included in JTF list;

† = labeled as check tag.

### Sex Check Tags Chosen by MTI Only

In three of 18 instances, MTI added sex check tags that were not in the original JTF list.

Citation #3 does not reference any specific population, but MTI suggests the check tag “Female†” (1) in addition to “Pregnancy†” (0), ranking both before “Humans†” (2) [21]. For Citation #11, MTI includes check tags “Male†” and “Female†”, and ranks “Male†” (1) three entries higher than “Female†” (4) [30].

For Citation #19, MTI includes both “Male†” and “Female†” as tags while the original indexer included neither [31]. The abstract describes a population of surgical residents (n=765) without specifying sex, a condition under which many human indexers would include check tags for both sexes. Consistent with all other citations for which MTI used both sex check tags, “Male†” (1) preceded “Female†” (2) by one rank in the Full Listing.

### Human Indexer Only Terms Not Found in Full Listing

Our study revealed a high average coverage rate of human-indexed terms in the Full Listing (89.75%). The coverage of human-indexed terms in MTI's Full Listing was 100% for 13 citations. In Citation #3, coverage was already 100% in the JTF list. A total of three terms across six citations processed by Interactive MTI were missing human index terms in the Full Listing. These terms are “Aged†” [29–30, 32–33]; “Breast Neoplasms” [24]; and “Receptor, Serotonin, 5-HT2A” [34].

In the four citations that missed “Aged†”, no mention was made of age in titles or abstracts. For Citation #9, MTI did not identify age-related tags in the JTF list [32]. Citation #11 refers to a “general population” in the abstract, and MTI identified “Adult†” and “Middle Aged†” [30]. The Full Listing includes “Adolescent†” (23) and “Aged, 80 and Over†” (25), which were used by the human indexer.

Citation #20 includes “Middle Aged†” (11) in its JTF list, and “Adult†” (12) and “Aged, 80 and Over†” (24) in its Full Listing, while Citation #12 has “Middle Aged†” (12) in its Full Listing [29, 33].

Citation #1 is about the early detection of cancer and includes the word “breast” in “screening recommendations for breast, colorectal, prostate, and cervical cancers” [24]. MTI suggests “Uterine Cervical Neoplasms” (3) and “Colorectal Neoplasms” (7) in the JTF, and “Prostatic Neoplasms” (16), “Colonic Neoplasms” (25), and “Rectal Neoplasms” (28) in the Full Listing. The term “Breast Neoplasms” is not included.

Citation #10 includes “Receptors, Serotonin, 5-HT3” (20), “Receptors, Serotonin, 5-HT1” (27) and “Serotonin” (1) in the Full Listing, but did not include Receptor, Serotonin, 5-HT2A [34].

### MTI Synonym Terms in Relation to Human Terms

For 19 citations, MTI used a term synonymous with one in the human indexer list, as summarized in Table 6. Two terms are considered synonymous when they are within two levels of each other in the MeSH tree. A “broader term” indicates that MTI chose a concept less specific than the human indexer; a “narrower term” indicates that MTI chose a more specific concept, and an “equivalent term” indicates that it chose an equivalent concept in specificity. Two synonyms are considered equivalent when one is listed as an entry term or previous indexing term for the other. MTI often chose the narrower term available but was inconsistent in doing so.

## DISCUSSION

Several findings in this study warrant further investigation, and can be summarized as follows: 1) the MTI assigned more terms and used terms more accurately for citations in the higher-JIF group; 2) MTI tended to rank “Male†” more highly than “Female†” and may omit “Aged†” check tags; 3) MTI may select more appropriate or more specific synonyms than human-indexed terms, but it was inconsistent in its use of terms with the highest level of specificity when describing some concepts.

### MTI Indexing for Higher and Lower JIF Journals

Overall, MTI assigned more terms and used them with more precision for citations from higher JIF journals than lower JIF journals. This is not due to the JIFs themselves, which Interactive MTI does not consider, but due to the tendency of general or popular clinical areas of biomedicine to have higher JIFs than allied health or specialized domains.

While the number of terms in a Full Listing varied depending on subject matter and text length, the number of terms included in the JTF list was based on the confidence scores. The threshold for inclusion in the JTF is unknown, as terms excluded are given MTI scores of 0 and -1 on the Full Listing. A short JTF list indicates that MTI deems fewer index terms appropriate for the text based on its confidence scoring.

The variance in MTI's scoring based on journal subject areas is worth scrutiny. Over time, the emergence of citations tagged with unrelated or distant index terms will affect searching accuracy. Reducing the precision of MeSH terms applied to any Medline record may translate into more work for searchers, who will have to create filters and workarounds to find the relevant Medline records. The omission of index terms, even temporarily, may mean that relevant citations will not be found.

As the high degree of human-indexed term coverage in MTI's Full Listing shows, its problems do not pertain to term retrieval but more to the ranking of retrieved terms. Based on the trends we observed, and upon closer examination of Citations #3, 14, and 17, it appears that relevant terms with lower rankings in the Full Listing are often terms denoting non-medical or allied health topics. This has far-reaching implications for qualitative, nursing, and social science research.

Citation #17 in particular exemplifies how MTI can misinterpret word meanings. MTI indexed Citation #17 with the term “Attention”, which is defined by the MeSH Browser as “the act of heeding or taking notice or concentrating” [35]. Neither the abstract nor the paper's full text covers this concept. The word “attention” appears in the phrase “[t]he concept of nursing practice models–shared governance–has attracted the attention of nursing administrators in the last decade…,” but MTI interpreted “attention” to mean “take interest” or “take notice”. The irrelevant term was included, while the key headings were not.

### Check Tag Problems

Reports from the NLM suggest that MTI frequently missed or misused check tags [2–3], which may be due to abstracts not clearly describing their study population. This may also reflect gender bias in the biomedical literature, with clinical trials prioritizing male participants [36]. There is an inherent gender bias in rankings for the check tag “Male†” over “Female†” when MTI identifies “Humans†” as the main tag, and where populations are not well-defined. These issues raise concern about MTI's use of titles and abstracts to generate terms for these tags. NLM has said that MTI will search the full text of papers in the future [1], but we could not find an estimated start date.

In this study, MTI made some unjustified sex check tag choices that human indexers did not make, see Table 5. For Citation #3, it is unjustified to leave out “Male†” and to rank “Female†” before “Humans†”, but the choice to include the check tag is logical, as the abstract references “eclampsia” and “pre-eclampsia” [21]. MTI is consistent at adding the check tag “Female†” when it identifies pregnancy-related conditions, as described in its Processing Flow document [14]. However, it may prescribe too much weight to “Female†” in certain instances. For Citation #11, MTI includes check tags “Male†” and “Female†”, and it ranks “Male†” (1) three entries higher than “Female†” (4). This is a poor choice, as the title and abstract are about evaluating a Woman Abuse Screening Tool, and there is no reference to men at all [30].

**Table 5.** Comparison of MTI and human check tags in three citations where MTI added additional sex check tags

Citation Number	MTI check tags	Human check tags
3	Pregnancy [0]; Female [1]; Humans [2]	Humans
11	Humans [0]; Male [1]; Adult [2]; Middle Aged [3]; Female [4]	Adult, Female, Humans, Middle Aged
19	Humans [0]; Male [1]; Female [2]	Humans

**Table 6.** Relationships between MTI terms and their synonymous human-indexed terms

Term Relationship	MTI Term	Human-Indexed Term
	Cell Transplantation	Islets of Langerhans Transplantation*
	Colorectal Neoplasms	Colonic Neoplasms Rectal Neoplasms
Broader MTI Term	Diabetes Mellitus	Diabetes Mellitus, Type 1*
	Methylation	DNA Methylation
	Mutation	Frameshift Mutation*
	Probability	Odds Ratio
	Sodium Chloride	Sodium Chloride, Dietary
	*Acute Lung Injury*	Lung Injury
	Blood Glucose Self-Monitoring	Monitoring, Physiologic
	Bone Plates	Internal Fixators*
	*Coronary Restenosis*	Coronary Disease
Narrower MTI Term	Delayed Action Preparations Drug Carriers	Drug Delivery Systems*
	*Mandibular Osteotomy*	Oral Surgical Procedures*
	Polyethylene Glycols	Polymers*
	*X Chromosome Inactivation*	Dosage Compensation, Genetic*
	Fibrinolytic Agents	Antithrombins*
Equivalent Terms	*Neointima*	Tunica Intima
	X Chromosome	Dosage Compensation, Genetic*

Terms that were entered into MeSH after the year 2000 are italicized.

With regards to age check tags, MTI omitted “Aged†” four times, making it the most consistent term omission in this study. Omitting “Aged†” is problematic as it leaves out the age range 65-79. While “Adult†” covers ages from 19+, it is not as useful as specifying age ranges, as searchers who use the “Aged” filter only may unintentionally filter the article out. Considering the large span of the “Aged†” check tag, this consistent omission may pose problems in information retrieval for age-specific and population-specific searches.

Only two human-indexed main headings were entirely omitted by MTI, but they were considerable omissions. The omission of “Breast Neoplasms” from Citation #1 is odd, as “Breast Neoplasms” is listed alongside several other neoplasms in the same sentence [24]. The omission of “Receptor, Serotonin, 5-HT2A” in Citation #10 is likewise unusual, as MTI identified two other serotonin receptors in its Full Listing and ranked “Serotonin” (1), a major heading by the original human indexer, at the top of the JTF [34]. The abstract refers to three types of 5-HT receptors. These are examples of MTI's shortage of discernment, and its potential inconsistency around locating precise terms.

### Specificity of MTI Terms

In nine instances, MTI was able to identify narrower terms than those used by the original human indexers. While this affirms MTI's capacity to reference and retrieve from the Unified Medical Language System (UMLS) and MeSH, there were omissions of main headings and check tags from the Full Listing. Further investigation is required to identify possible factors contributing to the omission of main headings.

The choice to use broader terms may be related to an insufficient description in the abstract to indicate that a concept should be assigned a more specific term. NLM's Medline Indexing Online Training Course instructs indexers to “[a]lways check for the most specific term” [37]. In all nine instances, MTI's choice of terms was correct but not the most specific. When few narrower terms are available, such as “Sodium Chloride, Dietary” for “Sodium Chloride”, the choice of a broader term may not affect searching. However, where numerous distinct narrower terms are available, such as “Frameshift Mutation” for “Mutation”, the narrower term is more specific and probably more accurate.

Generally, terms in the narrower and equivalent lists are accurate choices that offer more specificity compared to the original index terms. Interactive MTI has the advantage of two decades of improvements made to the MeSH vocabulary itself and draws on the most current MeSH data from 2022.

## RECOMMENDATIONS

Some solutions NLM has proposed to address MTI's shortcomings include refining Learning to Rank algorithms, improving automatic check tag generation for specific journals or subjects, and establishing appropriate cut-off levels for the inclusion of terms [3]. These methods seek to make MTI more autonomous and accurate. MTI may also benefit from using more diverse training data representing varied populations and subjects to reduce biases. In our view, these improvements are not equivalent to including more expert human indexers. NLM may wish to consider incorporating a greater extent of human curation for citations from under-represented fields and terms that are challenging for machines to predict [5].

Implementation of MTI as an automated indexing tool will bring changes to familiar indexing patterns. In comparison to human indexing, MTI may use higher or lower numbers of terms for some subjects and favor broader or narrower terms. Unless it is improved, reliance on MTI as a fully automated tool may compromise the integrity, precision, and utility of the MeSH thesaurus. Further, the MTIA may result in widespread, erroneous indexing patterns that contradict the original definitions of MeSH terms, thus diminishing the value of MeSH definitions.

We recommend that librarians continue to assess the impact of automated indexing of biomedical literature. This includes regularly performing keyword searches in PubMed in combination with MeSH vocabularies to optimize search sensitivity. For many librarians, this has now become standard practice, but searchers who have not adopted this practice may wish to develop and test new search filters and evaluate index terms more closely in the future. Meanwhile, librarians can report MeSH anomalies, indexing errors and biases to NLM's Support Center; a good example of this is the recent librarian campaign against MeSH terms that were deemed racist [38].

For those seeking to publish in a Medline-indexed journal, we recommend using words in the title and abstract fields that are highly descriptive. MTI assigns more weight to keywords in the titles of papers [16] and performs better when abstracts follow a structured format [11]. It has also been found to perform poorly when metaphors are used [39]. It may be useful to input a manuscript's title and abstract into a free MTI tool such as Interactive MTI or MeSH on Demand to test possible index terms. For further guidance on making papers more descriptive and findable in Medline, authors should consider speaking to a qualified medical librarian.

NLM's move to fully automated indexing of Medline has not been widely publicized, and there is a lack of publicly available data on MTI's recent performance. In the spirit of openness and transparency, we recommend therefore that NLM provide the most recent MTI to health sciences librarians for testing purposes, and to ensure future research on automated indexing of Medline can be accurately replicated. Further, health sciences librarians may want to consider gathering user feedback, sharing resources with each other to educate users on automated indexing, and using this information in Medline instruction. Future studies should enlist the expertise of human indexers and librarians for qualitative analysis of Medline indexing. Experienced biomedical indexers can offer insight into the manual indexing of papers and the implications of automated processes on efficient, effective subject analysis over time.

## LIMITATIONS AND FUTURE DIRECTIONS

Our study sample is small and not generalizable. However, despite the small sample, any differences we observed between citations in higher and lower JIF journals in the Interactive MTI are likely underestimating not overestimating effects, as most of the journals included in AIM are still core to clinical medicine. We acknowledge that the JIF may be an unreliable measure of a journal's impact and relevance [40]. Further, one citation could never be representative of the journal in which it was published. Future studies should sample a larger pool of journals and papers, based on subject areas, to ensure comparisons in automated indexing in different fields.

We acknowledge that our findings are based on the MTIFL of 2011, which NLM discontinued in 2021, rather than the current MTIA [39]. Similarly, comparing the Interactive MTI to human indexing done more than twenty years ago may not be an accurate reflection of indexing today. As biomedical research evolves, indexing standards and practices will vary, and many indexers agree that no single set of index terms will ever serve as a perfect standard [41–42]. Our goal in this study was to use a range of examples to illustrate potential issues with automated indexing in Medline, and to do so as the NLM completes this landmark transition.

## Data Availability

All data generated in this study are available in the Open Science Framework at https://osf.io/4k69q/.

## SUPPLEMENTAL FILES

**Appendix A:** List of sampled journals, their subject areas, and their 2020 journal impact factors
