# Supplementary material for: Automated indexing using NLM's Medical Text Indexer (MTI) compared to human indexing in Medline: a pilot study
Source: J Med Libr Assoc. 2023 Jul 10;111(3):684–94. doi: 10.5195/jmla.2023.1588 (PMC10361558; doi:10.5195/jmla.2023.1588)
Supplement: Supplementary file 1 — Appendix A: List of sampled journals, their subject areas, and their 2020 journal impact factors [file jmla-111-3-684-s01.pdf]

Appendix A. List of sampled journals, their subject areas, and their 2020 journal impact factors

Code	Journal	Subject Area	JIF (2020)
1	CA: a cancer journal for clinicians	Oncology	508.702
2	New England journal of medicine	Medicine, general & internal	91.253
3	Lancet (London, England)	Medicine, general & internal	79.323
4	JAMA	Medicine, general & internal	56.274
5	Circulation	Peripheral vascular disease; cardiac & cardiovascular systems	29.69
6	Annals of internal medicine	Medicine, general & internal	25.391
7	Journal of the American College of Cardiology	Cardiac & cardiovascular systems	24.093
8	Blood	Hematology	23.629
9	Gut	Gastroenterology & hepatology	23.059
10	Gastroenterology	Gastroenterology & hepatology	22.682
11	Journal of family practice	Primary healthcare; medicine, general & internal	0.493
12	Southern medical journal	Medicine, general & internal	0.65
13	Clinical pediatrics	Pediatrics	1.168
14	Nursing clinics of North America	Nursing	1.208
15	Journal of laryngology and otology	Otorhinolaryngology	1.469

16	Annals of otology, rhinology, and laryngology	Otorhinolaryngology	1.547
17	Journal of nursing administration	Nursing	1.737
18	Medicine	Medicine, general & internal	1.889
19	Journal of oral and maxillofacial surgery	Dentistry, oral surgery & medicine	1.895
20	American journal of physical medicine & rehabilitation	Rehabilitation; sport sciences	2.159
